# Longitudinal Trends in Childbirth Practices in Ethiopia

**DOI:** 10.1007/s10995-017-2279-y

**Published:** 2017-02-08

**Authors:** Julianne Weis

**Affiliations:** Anthrologica, 408 Cedar St NW #G, Washington, DC 20012 USA

**Keywords:** Ethiopia, Institutional birth, Birth choices, Intergenerational study

## Abstract

*Objectives* This study examines the influence of women’s birth practices on their daughters’ location of childbirth in Ethiopia, investigating the importance of intergenerational patterns of care on contemporary birth practices. *Methods* A qualitative survey of women aged 60 and over in three cities in Ethiopia. *Results* Nearly all first generation women gave birth at home, but the majority of their daughters give birth in facilities. Perceptions of childbirth practices among both women and their daughters have shifted towards facility births, despite the prevalence of home birth in the previous generation. *Conclusions* Birth culture has experienced a profound shift in Ethiopia within one generation, especially in urban areas, where health facilities are more easily accessible. Older generations of women have positive attitudes towards facility birth, and can help influence their daughters to give birth with medical assistance. This aligns with both national and global maternal health policies which promote safe motherhood through facility birth.

## Significance

The influence of family members on choices of medical care at the time of childbirth has been well documented in the literature. In Ethiopia, where just 28% of women give birth with a trained medical assistant, there is considerable evidence on the importance of family dynamics in determining birth location. This study illuminates these dynamics by presenting a historical context of intergenerational birth choices, analyzing the connection between a mother’s choice of birth location and her daughter’s. This is significant in helping chart the complex reasons for changing practices of childbirth in Ethiopia, a country with one of the highest maternal mortality rates in the world. The findings can help policy-makers and health-workers understand the historical context and community influences which determine contemporary birth practices.

## Introduction

At 420 deaths per 100,000 live births, Ethiopia has one of the highest rates of maternal mortality in the world (WHO 2014). To combat this high rate, the Ethiopian government has encouraged women to give birth in health facilities, where they can receive emergency medical assistance if necessary.

Despite this policy, Ethiopia currently has one of the lowest rates of facility births in sub-Saharan Africa: by latest estimates, just 28% of births are attended by a trained professional. The rates of skilled attendance at birth vary greatly by location, from 80% in urban areas, to 21% in rural areas (CSA and ICF 2016). In remote areas where facilities may not be available, community-level health extension workers (HEWs) have also been asked to help assist deliveries, though actual rates of HEW-assisted deliveries are minimal, at <1% (CSA and ICF 2012).

Numerous studies have investigated the reasons behind discrepancies in skilled attendance at birth, both in Ethiopia and other low-income countries. Distance to facilities, quality of care, financial barriers, and level of education of the woman have all been cited as primary determinants of birth location (Crissman et al. 2013; Gabrysch and Campbell 2009; Giacaman et al. 2006; Mpembeni et al. 2007; Mekonnen 2013; Moyer and Mustafa 2013; Teferra et al. 2012; Thind et al. 2008; Tsegay et al. 2013). In addition, the influence of both cultural beliefs and family members have been seen as primary indicators of skilled attendance at birth (Fikre and Demissie 2012; Kabakyenga et al. 2012; Mekonnen and Mekonnen 2003). In Ethiopia, there is a common-held belief that medical services are simply unnecessary at the time of delivery, so state-sponsored services, both clinics and HEWs, are not sought after (Shiferaw et al. 2013). These beliefs can be transmitted across generations, and reinforced by older family members.

While the influence of culture and family beliefs is well acknowledged in the literature, intergenerational patterns in the management of childbirth are rarely studied. This article presents findings from an intergenerational survey of women in Ethiopia, investigating the influence of a mother’s birth practices on her daughter. In a study on improving skilled birth attendance in developing countries, Van Lerberghe noted that “if we are serious about reducing global disparities in maternal death, we must address the social processes and relations that restrict the choices women make regarding their bodies and their lives” (Van Lerberghe et al. 2014). There are numerous social factors which determine a woman’s birth practices, including the influence of elderly relatives. Cultures of birthing are transformed across generations, with the urging or discouragement of family members. 

Studying the relationship in birth practices between generations helps us examine the ways in which global policies may fall short in broaching local practices. While the mandate to “think globally, act locally” is often cited in relation to maternal health, the fixation on facility birth in Ethiopia has led to a very slow and uneven progression of rates of skilled attendance at birth (Jaffre and Suh 2016). This study helps examine some of the limitations and enabling factors within local social relationships in Ethiopia to improve safe motherhood via facility birth, demonstrating the connection between geographic access to facilities and cultural shifts towards institutional delivery.

## Methods

A qualitative survey was performed in three cities and towns in Ethiopia: Addis Ababa, Gondar, and Jinka. The three sites were chosen because of their diversity in size, infrastructural capacity, and geography. Addis Ababa is the capital city of Ethiopia, and has a high density of health facilities. Gondar is a mid-sized town, with a large teaching hospital and numerous health centers. Jinka is a small town with one rudimentary hospital and health center, first opened in the mid-1960s.

The study made use of purposive sampling. In each site, the author made contact with local health centers and staff. Health extension workers helped identify relevant participants in their catchment area to be included in the study. The criteria for inclusion was gender and age: all participants in the study were women with an approximate age of 60 and over. In each town, we deliberately selected multiple *woreda* and *kebele* (administrative terms for district and neighborhood in Ethiopia) that represented varied ethnic, religious, and income groups. In this way, a variety of socio-economic groups were consulted, to identify variations in health-seeking behaviors based on demographic background. The age of second-generation women varied considerably, from 20 to 60, given the early age of childbearing for Ethiopian women.

In Addis Ababa, 80 women were interviewed in three different *woreda*. In Gondar, 15 women were interviewed in three different *kebele*. Last, in Jinka, 10 women were interviewed in two different *kebele*. The differences in numbers of women between the towns reflects the varied population densities of the three study sites, and a rudimentary sampling of women based on town population. While Jinka’s overall population is much smaller than Gondar, limitations in study resources meant that only 15 women were interviewed in Gondar. The diversity of *kebele* sourced in Gondar saw a range of responses, however, and it was felt that the sample size was still indicative of the urban population.

The majority of Ethiopians reside in rural areas, but this study was conducted solely in urban zones. Given the constraints of the study, this was a deliberate decision to be able to find a high volume of respondents within an easily accessible area. Further, because the study was primarily focused on elderly women, there was a recognition that many of the women interviewed would have lived previously in rural areas. This was especially the case in Addis Ababa: 70% of respondents had given birth in rural areas when they were younger, and only moved to the city later in life. As a retroactive study, the urban sampling was thus able to capture practices in both urban and rural areas.

The interviews were conducted in Amharic and Oromifyia. The author was introduced to the women by the local health extension worker, and conducted the interview through a translator with a standard semi-structured questionnaire.

Initial ethical approval for the study was obtained through the University of Oxford, Addis Ababa University, and Gondar University. Informed consent was then obtained from each woman at the start of the interview.

## Results

In all sites, the majority of first generation births took place at home, attended either by traditional birth attendants, neighbors, or relatives. These births took place between ca. 1955–1990, when health services, even in Addis Ababa, were rudimentary. In Addis Ababa, 54 women gave birth to the majority of their children at home, with 25 giving birth in health centers or hospitals. One woman had not given birth to children, but instead adopted five children that her husband had had with a previous wife.

In Gondar, 11 first generation women gave birth at home. For three of those women, they were attended by government-trained community nurses at home during the labor. The remaining four women gave birth at either a health center or hospital.

In Jinka, nine first generation women gave birth to the majority of their children at home, and one woman gave birth primarily at the health center.

Interestingly, there was evidence of longitudinal changes in place of birth even within the span of the woman’s life: I have counted the ‘place of birth’ above as the most common place of delivery, whether at home or hospital, as nearly half of all women interviewed alternated between both locations during their reproductive history.

Parity was high among women, with a median of 7 births across the sample group. Women began giving birth early in life, and continued well into their late thirties and early forties. This meant that several women’s reproductive history spanned multiple governing regimes in Ethiopia: women may have started to give birth to their first children in the Haile Selassie regime, when health services were extremely limited, and then continued having children late into the Derg regime, when services were expanded.

For 33 women in Addis Ababa, while their first births were mostly at home, they had a later birth in a health center or hospital. A similar trend was noted in Gondar (6 women), and Jinka (5 women).

Intergenerational changes were even more profound: for just 12 women in Addis Ababa who had given birth at home, their daughters also gave birth at home. For the remaining 42 women who had given birth at home, their daughters gave birth at health centers or hospitals in the capital city. There were no women who had given birth in a facility whose daughters gave birth at home. There was therefore a clear continuation pattern between facility birth across generations: if the woman gave birth at a health facility, her daughter did the same.

In Gondar and Jinka, aside from one woman in Jinka, all reported their daughters giving birth in facilities. The one woman whose daughter gave birth at home in Jinka had herself given birth at home. Otherwise, because the rest of the second generation of women had facility births, there was no predictive pattern of continuation in the place of birth between the mother’s and daughter’s place of birth.

Religion was not a predictive factor in either first or second generation births, nor was the use of ante-natal care. On the contrary, while many first and second generation women had received at least one ante-natal care visit during pregnancy, there were still high rates of home delivery. This is consistent with other studies from Ethiopia that show that ante-natal rates are not correlative to facility birth (CSA Ethiopia and ICF 2016; Shiferaw et al. 2013).

Social class and income level were strong predictive factors of birth location. Farmers and laborers were considered lower class, while any profession requiring an education, either for the woman or her husband, including teachers, bankers, and government workers, were considered middle and upper class. While there were some lower class women who gave birth in facilities, it was universal for middle and upper class women to have facility births, both in the first and second generations.

## Discussion

In interviews, first generation women expressed a variety of opinions on the necessity of medical assistance at the time of childbirth. The majority of women saw no need for intervention in their own births, but some admitted the relevance of medical assistance for their daughters. All middle and upper class women expressed a strong preference for facility births for themselves and their daughters, while responses among lower class women were influenced by personal experiences with obstetric complications, shifts in class and social standing across generations, and geographic proximity to services.

For a handful of women in Addis Ababa whose daughters had given birth at home, there was an active resistance to medical intervention at the time of childbirth. One woman, a poor laborer, explained that she would only allow her daughter to go the hospital if there were male doctors present, stating that “women are not good at medicine, only men are good.” This woman’s daughter, present for the interview, also interjected, explaining that she had given birth to three children at her home in central Addis Ababa because she is afraid of the hospital. Her mother replied that for her own births, there were no complications, so she saw no need for medical intervention. At the same time, living in the city center, less than one kilometer from a health center, she insured that all her children were brought to health centers for necessary immunizations, and would resort to state medical services whenever her children were ill.

These observations help illustrate the complex and varied relationship between Ethiopian women and contemporary health services. While both mother and daughter conceded that medical services were necessary in some instances, for childbirth, they were better left on their own at home. Distance to facilities was not an issue, but gender biases and fear of health staff were influential over the women’s decisions to seek services at the time of childbirth. The lower social standing of the women, neither of whom had had a formal education, also influenced their perception of health services.

In contrast to this woman and her daughter, there were several lower class first generation women who, while happy to have given birth themselves at home, openly encouraged their daughters to make use of medical facilities. The reasonings varied: for the majority of these women, they had lived in remote rural areas when they gave birth, and facilities were simply not an option for them. They understood that now, residing in either a town or city, less than three to five kilometers from a hospital, their daughters could more easily travel to a clinic or hospital at the time of delivery by walking, taking a taxi, or borrowing the car of a neighbor. For these women, the issue was one of physical proximity and access to services. Even if they had not had personal experience with obstetric complications, they had clearly internalized the state policy rhetoric which argues for the greater safety of facility delivery. There was also often a shift in class between generations: while the mothers were all poor farmers or laborers, having moved to the city later in life, their daughters had all received an education and were part of a burgeoning urban middle class. This influenced their receptivity to state policies promoting facility birth.

This pattern was especially true of the women in Gondar and Jinka, where distances to facilities were even shorter, often walking distance. For all but one of the women interviewed in Gondar and Jinka, their daughters gave birth in town health facilities. For many of these women, the fact that their daughters had facility births was also seen as a signal of improved social status: they now lived in the town, and were proud to make use of the urban services now available, including government schools and health clinics. There was a sense that this was the right course of action, in contrast to their previous practices of home birth.

When discussing the decisions of their daughters to give birth at a facility, many women also acknowledged that this was simply a generational change. Before, as one poor farmer said, “we just gave birth.” Another woman explained that “we didn’t have a choice, as a culture”: home birth was simply how it was done for the vast majority of Ethiopian women in previous years. This was a reflection not only of the extreme paucity of health services before 1990, but also a cultural reflection of the normalization of childbirth. As one woman who worked as a traditional healer explained, while doctors were good, “so were traditional birth attendants.” They provided their own necessary services, and were well-qualified for the time.

In addition to citing historic cultural norms, many women mentioned that before, they were simply healthier than their daughters. The majority of first generation women were farmers, working long days in the field and eating a whole grain diet. To them, this made birth easier. Now, they believed that there were more diseases, and the urban lifestyle made their daughters less physically fit to deliver. One woman who had been a farmer claimed that in former times, “there were no diseases,” but now, everyone is “too afraid” of conditions like HIV. This made medical services essential, whereas before, they were largely unnecessary.

Given all demographic evidence of health and disease in Ethiopia before 1990, it is evident that there was not a golden age free of disease.^1^ Nevertheless, the nostalgia for the past may indicate a sustained preference for home remedies of care in times of birth, and potential reticence to adopt state policies promoting clinic birth. For those women who contrasted the health of the two generations, the majority had moved to the city later in life and seen a shift in class and educational levels among their daughters. While there were clear benefits to this shift, there was still an acknowledgment that home-based care options were a viable, and often preferable, option in matters of childbirth and postpartum care. This helps explain the perpetuation of home birth in Ethiopia, even in urban areas where facilities are accessible.

At the same time, the consistent reference to contemporary diseases and complications in childbirth is evidence of the strength of state-directed public health campaigns which encourage women to deliver in the hospital in the name of safety and health. Since 2000, government rhetoric in Ethiopia has been vigilant in promoting multiple public health initiatives, including HIV prevention and skilled attendance at childbirth. The repetition of official rhetoric demonstrates the ways in which state policies have largely changed the conversation on matters of childbirth for women across socioeconomic classes in Ethiopia, reinforcing the notion that facilities are a better alternative than home birth.

While the messaging is widespread, this did not mean that all first-generation women would actively encourage their daughters to deliver at a facility. When asked about the birth practices of their daughters, several lower class women interviewed would concede that the care at facilities was good only with a degree of reluctance. One lower class woman explained that while she had given birth at home, and she felt healthy and fit after the birth, she was astonished after her daughter gave birth at a hospital in Addis Ababa, as she was strong “immediately after birth.” The daughter returned from the facility “clean and healthy,” in contrast to the mother, who had to wait several weeks at home for birth injuries to heal on their own. These mothers previously did not think that giving birth in a facility was necessary, and were only convinced of its use after seeing their daughters give birth with success within the city hospitals.

## Conclusion

This study has described the generational shift from home to facility birth in Ethiopia. It is made clear that contemporary, urban women in Ethiopia are accustomed to giving birth in facilities, even if their mothers had given birth at home. The influence of first generation women’s reproductive histories and perceptions of medical services was evident in the birth practices of their daughters. Public health rhetoric on the risk associated with childbirth and the necessity of medical intervention were also clearly transmitted both to first and second generation women in the study.

This study is limited in that it discussed contemporary birth practices of solely urban or peri-urban women. Still, it has helped demonstrate that when physical distance to health facilities is minimized, women in Ethiopia are highly inclined to deliver in a hospital or health center. Even when personal family history indicates a cultural preference to give birth at home, women in urban areas of Ethiopia are choosing facility births over home-based traditions.

The results of this study also demonstrate that even when belief in certain cultural practices related to childbirth are strong, they can be aligned towards public health policies. While first-generation women were mostly happy with their own home births, they were comfortable with, and sometimes encouraging of the shift towards facility-based birthing for their daughters. This encouragement often came after their daughters gave birth in a facility, and they saw the safety and quality of care.

The arc of history in Ethiopia is bent towards institutionalized childbirth. In rural areas of the nation, more women will give birth in medical facilities if access to clinics is improved. The government has also encouraged health extension workers in rural areas to attend births at home, when facilities are not available, although the success of this initiative has been limited. Reluctance to use medical professionals and facilities among Ethiopian women is prominent, especially among lower class women, but at the same time, cultural norms on childbirth are shifting with the new generation. In the last 5 years, skilled attendance at birth has risen from 10 to 28%, with the greatest gains in urban areas. Once women in rural areas are granted the same level of access to medical facilities at the time of childbirth as those in urban areas, there is no reason to believe that their cultural predilections will not also shift towards medicalized services.

Two studies from Zambia and Malawi demonstrated that closer geographic proximity to high-level health facilities was associated with more frequent facility delivery (Gabrysch et al. 2011; Lohela et al. 2012). In order to see a nation-wide improvement in the number of women giving birth in facilities, wider access to quality health services is vital. This can come in the form of an improved number of clinics, or better accessibility of health extension workers and other community-level services. Further, engaging older generations of women in discussions on birth choices can help facilitate shifts in cultural predilections for maternal care.

## Footnotes

1 Vital statistics on Ethiopia before 1990 are scant, and often estimates. Still, it is estimated that half of children died before age five, and that maternal mortality was as high as 2,500/100,000 live births. Malaria, cholera, tuberculosis, pneumonia, and gastrointestinal diseases were also exceptionally common. See Kloos & Zein 1988.

## References

[CR1] Central Statistical Agency (CSA) Ethiopia and ICF. (2012). Ethiopia Demographic and Health Survey 2011.

[CR2] Central Statistical Agency (CSA) Ethiopia and ICF. (2016). Ethiopia Demographic and Health Survey 2016.

[CR3] Crissman HP, Engmann CE, Adanu RM, Nimako D, Crespo K, Moyer C (2013). Shifting norms: Pregnant women’s perspectives on skilled birth attendance and facility-based delivery in rural Ghana. African Journal of Reproductive Health.

[CR4] Fikre AA, Demissie M (2012). Prevalence of institutional delivery and associated factors in Dodota Woreda (district), Oromia regional state, Ethiopia. Reproductive Health.

[CR5] Gabrysch S, Campbell O (2009). Still too far to walk: Literature review of the determinants of delivery service use. BMC Pregnancy and Childbirth.

[CR6] Gabrysch S, Cousens S, Cox J, Campbell OMR (2011). The influence of distance and level of care on delivery place in rural Zambia: A study of linked national data in a geographic information system. PLoS ONE.

[CR7] Giacaman R, Abu-Rmeileh N, Wick L (2006). The limitations on choice: Palestinian women’s childbirth location, dissatisfaction with the place of birth and determinants. European Journal of Public Health.

[CR8] Jaffré Y, Suh S (2016). Where the lay and the technical meet: Using an anthropology of interfaces to explain persistent reproductive health disparities in West Africa. Social Science & Medicine.

[CR9] Kabakyenga JK, Ostergren P, Turyakira E, Pettersson KO (2012). Influence of birth preparedness, decision-making on location of birth and assistance by skilled birth attendants among women in South-Western Uganda. PLoS ONE.

[CR10] Kloos H, Zein ZA (1988). The ecology of health and disease in Ethiopia.

[CR11] Lohela TJ, Campbell OMR, Gabrysch S (2012). Distance to Care, Facility Delivery and Early Neonatal Mortality in Malawi and Zambia. PLoS ONE.

[CR12] Mekonnen Y (2013). Patterns of maternity care service utilization in Southern Ethiopia: Evidence from a community and family survey. Ethiopian Journal of Health Development.

[CR13] Mekonnen Y, Mekonnen A (2003). Factors influencing the use of maternal healthcare services in Ethiopia. Journal of Health, Population, and Nutrition.

[CR14] Moyer C, Mustafa A (2013). Drivers and deterrents of facility delivery in sub-Saharan Africa: A systematic review. Reproductive Health.

[CR15] Mpembeni R, Killewo JZ, Leshabari MT, Massawe SN, Jahn A, Mushi D, Mwakipa H (2007). Use pattern of maternal health services and determinants of skilled care during delivery in Southern Tanzania: Implications for achievement of MDG-5 targets. BMC Pregnancy and Childbirth.

[CR16] Shiferaw S, Spigt M, Godefrooij M, Melkamu Y, Tekle M (2013). Why do women prefer home births in Ethiopia?. BMC Pregnancy and Childbirth.

[CR17] Teferra AS, Alemu F, Woldeyohannes SM (2012). Institutional delivery service utilization and associated factors among mothers who gave birth in the last 12 months in Sekela District, North West of Ethiopia: A community-based cross sectional study. BMC Pregnancy and Childbirth.

[CR18] Thind A, Mohani A, Banerjee K, Hagigi F (2008). Where to deliver? Analysis of choice of delivery location from a national survey in India. BMC Public Health.

[CR19] Tsegay Y, Gebrehiwot T, Goicolea I, Edin K, Lemma H, San Sebastian M (2013). Determinants of antenatal and delivery care utilization in Tigray region, Ethiopia: A cross-sectional study. Journal for Equity in Health.

[CR20] Van Lerberghe W, Matthews Z, Achadi C, Campbell J, Channon A, de Bernis L (2014). Country experience with strengthening of health systems and deployment of midwives in countries with high maternal mortality. The Lancet.

[CR21] WHO, UNICEF, UNFPA, The World Bank and the United Nations Population Division. (2014). Trends in maternal mortality: 1990–2013.

[CR22] Yanagisawa S, Oum S, Wakai S (2006). Determinants of skilled birth attendance in rural Cambodia. Tropical Medicine and International Health.

